# Outpatient parenteral antimicrobial therapy (OPAT) in patients with cystic fibrosis

**DOI:** 10.1186/s12879-015-1019-4

**Published:** 2015-07-27

**Authors:** Maya Graham Pedersen, Søren Jensen-Fangel, Hanne Vebert Olesen, San deep Prataprao Tambe, Eskild Petersen

**Affiliations:** Department of Infectious Diseases, Aarhus University Hospital Skejby, Palle Juul jensens Boulevard 100, Aarhus, Denmark; Department of Pediatrics, Aarhus University Hospital Skejby, Palle Juul jensens Boulevard 100, Aarhus, Denmark; Department of Anestesiology, Aarhus University Hospital Skejby, Palle Juul jensens Boulevard 100, Aarhus, Denmark

**Keywords:** Home therapy, Intravenous, Complications, Peripheral venous line, PICC-line, PORT-A-CATH

## Abstract

**Background:**

To determine complications during outpatient parenteral antimicrobial therapy (OPAT) administrated through a peripheral venous line, PICC-line or PORT-A-CATH (PAC).

**Methods:**

Catheter related complications in patients with cystic fibrosis during OPAT were identified through a retrospective review of patient files supplemented by an interview.

**Results:**

In 64 treatment episodes with a peripheral venous line, 51 (79.7 %) used bolus injection and 13 (20.3 %) used infusion pump. 27 out of 51 (53.0 %) bolus injection episodes experienced complications, which required removal. None were observed for infusion pump treatments.

The infectious complications requiring removal of peripheral venous line were 9 out of 23 (39.1 %) for the PICC line and 11 out of 26 (42.3 %) for the PAC.

No anaphylaxis was observed during the OPAT treatments.

**Conclusions:**

Our data indicate that using an infusion pump to administer the antibiotic treatment minimized peripheral venous line complications. The frequency of complications leading to removal of the catheter is about the same for PICC-lines and PACs, but the average life-time of the latter is much longer. Allergic reactions are not a major problem.

**Electronic supplementary material:**

The online version of this article (doi:10.1186/s12879-015-1019-4) contains supplementary material, which is available to authorized users.

## Background

Cystic fibrosis (CF) is a genetic disorder caused by mutations in the CFTR gene, resulting in a multisystem disease dominated by pulmonary symptoms and the establishment of chronic pulmonary infections with bacteria, e.g. *Staphylococcus aureus*, *Haemophilus influenzae*, and *Pseudomonas aeruginosa*. The infections and the associated inflammatory responses are the most important causes of morbidity and mortality in CF patients today. The chronic inflammation of the lungs leads to bronchiectasia and lung fibrosis which eventually will lead to respiratory failure [1, 2]. Effective treatment of the pulmonary infections significantly reduces the deterioration in lung function over time and increases life expectancy significantly [2].

In CF patients, antibiotic treatments are delivered by three routes; intravenous, oral and as an inhaled aerosol [3]. Patients who exhibit significant symptoms of pulmonary exacerbation, e.g. fever and increased coughing, will require intravenous antibiotic therapy especially if infected with bacteria solely susceptible to antibiotics which can only be administrated intravenously [4]. In most instances lung function improves during the first few days of treatment, but antibiotic treatment for 10 to 21 days is usually required in order to achieve the highest possible reduction in pulmonary bacterial load [4].

The option of outpatient parenteral antimicrobial therapy (OPAT) [5] gives the CF patients an opportunity to continue their daily life with minimal disruption while undergoing antibiotic treatment essential for their quality of life. Furthermore OPAT reduces the risk of transmission of bacteria between CF patients and reduces isolation procedures at the hospital [6]. However, OPAT does come with the challenge of ensuring that the patient delivers the right amount of antibiotics at the right time without adverse reactions; Otherwise, the therapy may fail or be unnecessarily prolonged [2, 3]. The focus of this study was to estimate the frequency of the complications observed during and after OPAT through a peripheral line (Bectorn Dickinson, Franklin Lakes, NJ, United States of America) and through central lines (PICC-line and PORT-A-CATH (PAC) among CF patients followed at The Cystic Fibrosis Center West at Aarhus University Hospital, Denmark.

## Methods

### Study protocol

A retrospective review was performed on the 167 CF patients followed at the Cystic Fibrosis Center at Aarhus University Hospital, Denmark, identifying patients who received regular OPAT with peripheral or central intravenous lines (PICC-line and PORT-A-CATH (PAC)). OPAT is defined as the actual period in which the patients receive their antibiotics, and line insertion refers to the setting where the patients have their peripheral or central intravenous lines (PICC-line and PAC) in place regardless if treatment takes place. Complications accrued during line insertions life-time, which may have included multiple OPATs, were documented by reviewing the patients’ files. Since some CF-patients had more than one device implanted, the number of complications were normalized relative to individual line insertions and not treatments at such.

The study was part of the routine quality assurance program of outpatient antibiotic treatment, OPAT scheme in our department. According to Danish law, data from quality assurance schemes does not need approval by the scientific ethical committee if the data are anonymized before publication as is the case in this report.

### Patients

We included 60 CF patients who received antibiotic treatment using one or more of the following devices: a peripheral intravenous line in 2012 and 2013 (*n* = 64), a PICC-line in the period 2009 – 2013 (*n* = 23) and a PAC in the period 2005 – 2013 (*n* = 26).

### Questionnaire

The study used patient files combined with questionnaire driven patient interviews (33 patients, Additional file 1) to capture complications relating to the implanted lines leading to preterm removal of the catheter. The interview was essential because complications with the peripheral intravenous lines were not always registered in the patient files. Patients were initially contacted by e-mail and then by phone. After three messages on their answering machine no further attempts were made to contact the patients.

### Statistical analysis

A Mann–Whitney test was used to compare the life-time of the peripheral venous line.

## Results

### Peripheral intravenous line

A total of 95 line insertions were identified with the peripheral intravenous line and complications were documented by an interview. A total of 21 patients with peripheral intravenous line were interviewed covering 64 of the 95 line insertions in a two year period (2012 to 2013) and these 64 line insertions with the peripheral intravenous line have been included in this review. The age of the patients ranged from 3 to 27 years (median 18 years). The OPAT lasted between 7 and 25 days (median 14 days) corresponding to a total of 898 treatment days. The antibiotics were administered either by bolus injection or continuous administration by an elastomeric infusion pump. Out of the 64 line insertions with the peripheral intravenous line, 13 (20.3 %) used infusion pumps for their antibiotics treatment and the remaining 51 (79.7 %) used bolus injections. The antibiotics as with infusions were always tazocin (piperacillin/tazobactam )/sulfobactam whereas the antibiotics used with bolus administration were either meropenem, ceftazidin, cufuroxim or tazocin/sulfobactam. The most commonly used antibiotic was tazocin which was administrated to 48 % of the CF patients. Stratification by the life-time of line insertions of the peripheral line based on the bolus injection method showed that 21/51 (41.2 %) of the peripheral lines had complications with a life-time of the peripheral line of less than 5 days (Table 1), whereas all 13 OPAT using the infusion pumps had a life-time of the peripheral line of at least 5 days. The median life time of the infusion pumps were 7 days whereas the bolus injections had a median life time of 5 days (Table 1). However, the difference was not statistically significant (*p* = 0.242). The most common causes for replacement of the peripheral venous access were blockage or dislocation and pain while injecting (Table 2).

**Table 1:** Life time of the peripheral venous line

Peripheral venous line (type of injection)	Number of line insertions (%)	Life time of the peripheral venous line			Range (days)	Median (days)
		1–2 days (%)	3–4 days (%)	≥5 days (%)		
Infusion pump	13 (20.3)	0 (0.0)	0 (0.0)	13 (100.0)	5–7	7
Bolus injection	51 (79.7)	13 (25.5)	8 (15.7)	30 (58.8)	1–16	5
Total	64 (100.0)	13 (20.3)	8 (12.5)	43 (67.2)	1–16	7

**Table 2:** Peripheral venous line. Complications which resulted in replacement

Reason for replacements (*n*=52)	No. (%)
Infection	3 (5.8)
Pain while injecting	15 (28.8)
Blockage or dislocation	29 (55.8)
Fell out	5 (9.6)

### PICC-line

From 2009 to 2013, 23 line insertions with PICC-line were recorded. The age of the patients ranged from 9 to 32 years (median 22 years). The catheter life-time ranged from 1 to 201 days (median 17 days including planned removal after antibiotic treatment). In 15 (65.2 %) line insertions the PICC-line was scheduled for removal after completion of OPAT but 3 (20.0 %) of these line insertions were removed prematurely due to complications. In 8 (34.8 %) line insertions the PICC-line was kept in place after the end of OPAT but 6 (75.0 %) had to be removed subsequently due to complications (Table 3). In total nine (39.1 %) of the 23 implanted PICC-lines were removed due to complications (Tables 3 and 4).

**Table 3:** PICC-line complications

PICC line insertions (*n* = 23)	No. (%)	Complications causing removal (%)	Total treatment days	Range (Days)	Median (Days)
Removed when OPAT ended	15 (65.2)	3 (20.0)	160	1–28	14
PICC line kept in after OPAT	8 (34.8)	6 (75.0)	667	33–201	55
Total	23 (100.0)	9 (39.1)	827	1–201	17

**Table 4:** PICC line removals

Reason for removal (*n* = 9)	No. (%)
Infection	2 (22.2)
Pain	2 (22.2)
No return flow	1 (11.1)
Occlusion of the catheter	1 (11.1)
Dislocation	1 (11.1)
Line breakage	1 (11.1)
Patient asked for removal	1 (11.1)

### Port-a-Cath, PAC

In the period 2005 to 2013, 26 line insertions with PAC were recorded. The age of the patients ranged from 9 to 42 years (median 24 years). The life time for the PACs that has been removed ranged from 109 to 3,379 days (median 484). In the 15 patients currently having a PAC implanted the age of the implant ranged from 4 to 3,154 days (median 738). The PAC was removed due to complications in 42.3 % (Table 5) of the 26 line insertions. The complications leading to removal of the catheter were infections, cosmetic considerations, displacements of the catheter, occlusion of the catheter and one unknown cause of PAC removal (Table 6).

**Table 5:** Port-a-Cath, PAC, complications

Line insertions (*n* = 26)	No. (%)	Total treatment days	Range (Days)	Median (Days)
Removed due to complications	11 (42.3)	8320	109–2,279	484
Still have theirs in	15 (57.7)	18910	4–3,154	738
Total	26 (100.0)	27230	4–3,154	735

**Table 6:** Port-a-Cath, PAC, removals

Reason for removal (*n* = 11)	No. (%)
Infection	3 (27.3)
Cosmetic considerations	3 (27.3)
Displacement of the catheter	2 (18.2)
Thrombosis	2 (18.2)
Unknown causes	1 (9.1)

### General experiences with OPAT

Out of a total of 113 line insertions used in connection with antibiotic treatment 16 insertions gave rise to an allergic or toxic reaction with 14 (12.4 %) occurring during OPAT and 2 (1.8 %) at the initiation of the treatment in the hospital. The reactions were rash, fever or vomiting but no anaphylactic reactions were recorded. In Denmark the administration of antibiotics in the OPAT setting is performed by the patient or family members.

One part of the study looked at the CF-patients experiences with preparation and storage of their antibiotics. Only one patient was found to have a problem with the antibiotics taking 35 min to dissolve and there was one case where the drug crystallized in the infusion pump. The patients using infusion pumps found them very convenient because of the short preparation time prior to administration, less utensils needed to mix the medicine and consequently the procedure took up less space in the household and generated less waste.

Many CF-patients with a PICC-line or PAC for OPAT initially had a peripheral line. The primary reason for the patient to switch to a PICC-line or PAC for OPAT was the many problems they experienced with their peripheral lines such as frequent dislocations or blockages during treatment. Patients who chose PICC-line typically did not want a more permanent device like the PAC and the PICC-line gave them the possibility to remove the intravenous access immediately after their antibiotic treatment, which 15 (65.2 %) of the PICC-line users chose to do. Patients choosing PICC-lines also found them cosmetically more acceptable having the inserted catheter on the upper arm instead of the peripheral line on the dorsum of the hand. Two out of 23 (8.7 %) PICC-line insertions were thought to be so painful that the patient did not want a PICC-line implanted again and therefore wanted to use the peripheral venous line instead.

## Discussion

With OPAT usually lasting between 10–21 days [4] it is most convenient for the CF-patient if their peripheral venous line does not need to be changed too often. Therefore the CF-patients keep their peripheral venous lines until they experience complications, e.g. pain or blockage before replacing it. In this study we found that the median life-time of the peripheral venous line using an infusion pump was longer (7 versus 5 days) compared to using a bolus injection. This could suggest that the more constant flow of antibiotics with the infusion pump might be less traumatic for the vein leading to a prolonged life-time of the peripheral venous line. Nevertheless, the median life-time of the peripheral venous line was found to be at least 5 days. This is longer than the recommended replacement every 3–4 days by Cheung et al. [7] and support observations by Lai [8] suggesting that the peripheral venous line can stay in for a longer period of time given the right circumstances. Based on the research done by Lai [8] a peripheral line lasting less than five days during OPAT was defined as a complication and our results also support that peripheral lines should last at least 5 days. The peripheral venous lines are used by CF-patients for antibiotic treatment and this might have a positive effect on the use-life of the peripheral line (less risk of infection). Thirteen of 64 (20.3 %) line insertions only lasted 1–2 days and 8/64 (12.5 %) lasted 3–4 days resulting in a total of 32.8 % chance of complications necessitating the peripheral venous line to be changed before five days. However, none of the patients using infusion pumps on peripheral lines experienced complications. Patients should consider an alternative if their peripheral venous line has to be changed too often or should consider changing administration method since our research indicates that use of an infusion pump seems to improve the life time of the peripheral venous line. The alternative to a peripheral venous line could be a more permanent solution like a PICC-line or a PAC.

A total of 23 line insertions with the PICC-line were registered over a period of 4 years. The life-time use of PICC-lines is reported be used up to 6 months [7]. Tolomeo *et al.* [9] found complications which lead to removal in 18 (29.5 %) of the implants with the median life-time of 15 days. This compares to our study which showed that 9 (39.1 %) of the PICC-lines were removed due to complications after median life-time of 17 days. However, most of our patients did not have complications during the actual OPAT and only 20.0 % were removed because of complications during OPAT. The majority 75.0 % of complications occurred while the catheters remained in place after completion of OPAT.

The PICC-line is a suitable solution for patients not wanting a permanent PAC. PICC-lines can be removed after completed OPAT and 65.2 % chose to do this. Out of the 34.8 % PICC-lines where the patients elected to keep the line inserted after completed OPAT the median life time was 55 days. However, the majority of the PICC-lines had to be removed due to complications. Our experience with the PICC-line does not indicate that it is advantageous to keep the catheter inserted after completed OPAT. Some patients do not consider the PICC-line solution because they have heard that the PICC-line insertions are painful but of the 23 line insertions only 2caused the patient to not consider having a new PICC-line inserted.

Out of a total of 26 PACs implanted over a period of 8 years, 11 (42.3 %) were removed due to various complications with infection being the most common reason for removal. The extent of catheter removals correspond to that found by Munck et al. [10] who reported that 37 % of the catheters were removed due to complications. Compared with the study by Munck et al. [10]*,* the infection rate was approximately the same, however Munck et al. [10] reported a higher percentage of catheter occlusions 48 % compared to 18 % in our study. As a long term solution the PAC could be a viable solution with the total median life-time of the PAC was in our study found to be at least 735 days with 15 (57.7 %) patients still having their PACs in place. In another study the PAC was reported to stay intact for an average of up to 5 years [11] and in our study we currently have a patient who has had the PAC for 3,154 days (almost 9 years).

Patients who do not consider the more permanent solution explain that they are scared of thrombosis or occlusion leading to more serious conditions. The data from our study show that only 2/26 (7.7 %) of the PAC were removed due to a thrombosis and 1/23 (4.3 %) of the PICC-lines were removed due to occlusion of the catheter. The data indicate that the risk of a thrombosis or occlusions of the catheter with PAC or PICC-line are relatively low.

## Conclusions

Aiming for the best possible quality of life for the CF-patients, OPAT was introduced and the goal is to minimize the complications experienced during OPAT. This review quantitates the complications of different types of line insertions. Which line insertion to choose will also depend on how permanent the patient wants the device to be as well as the consideration of how often they are in need of antibiotic treatment [7]. OPAT is very important to the patients’ quality of life and everyone who was interviewed was glad that they had the choice of OPAT. They all wanted it to be successful to avoid being confined to the hospital in periods where intravenous antibiotic treatment is needed. This been said patients do experience problems and because antibiotic treatment is such an important part of their life the amount of complications have a significant impact on the time and energy they spend on their line insertions. Therefore, with a better understanding of the complications experienced with the different type if line insertions and during OPAT it is hoped that OPAT can be further improved.

Limitations of this study is that it was conducted as a retrospective study reviewing complications experienced in the OPAT setting over a period of limited duration and that the sample size in the study was limited to 60 patients from only one of the two CF centers in Denmark.

## Additional file

Additional file 1:
**Phone questionnaire.**
