# Supplementary material for: Outpatient parenteral antimicrobial therapy (OPAT) in patients with cystic fibrosis
Source: BMC Infect Dis. 2015 Jul 27;15:290. doi: 10.1186/s12879-015-1019-4 (PMC4515313; doi:10.1186/s12879-015-1019-4)
Supplement: Additional file 1: — Phone questionnaire. [file 12879_2015_1019_MOESM1_ESM.docx]

**Additional file 1 Phone questionnaire**

I

1. How many antibiotic intravenous treatments have you had in 2013 and in 2012?
2. Have you followed the guidelines form The Cystic Fibrosis Center West at Aarhus University Hospital Skejby?
3. If it has been necessary to change the peripheral intravenous line during the antibiotic intravenous treatment, how many times do you in general think it was changed?
4. What was the most common reason for the peripheral intravenous line to be changed?
5. Have you had bolus injection or infusion pump?
6. Have you experienced any problems with mixing the medicine if that has been necessary?
   1. If yes, do you think that it takes a long time to mix?
7. Have you had problems with the medicine storage?
   1. Do you think that it takes op a lot of space?
8. What did you have before?
   1. Why did you change to PICC line?
9. Have you followed the guidelines form The Cystic Fibrosis Center West at Aarhus University Hospital Skejby?
10. Have you had bolus injection, infusion pump or bolus?
11. Have you experienced any complications?
    1. Infection
    2. No return flow
    3. Occlusion
    4. Pain
    5. Other problems
12. Have you experienced any problems with mixing the medicine if that has been necessary?
    1. If yes, do you think that it takes a long time to mix?
13. Have you had problems with the medicine storage?
    1. Do you think that it takes op a lot of space?

Questionnaire for CF-patients who have used PICC line in 2009-2013 for their antibiotic intravenous treatment

Questionnaire for CF-patients who have used the peripheral intravenous line in 2012- 2013 for their antibiotic intravenous treatment

Questionnaire for CF-patients who have used the Port-a-Cath in 2005- 2013 for their antibiotic intravenous treatment

1. What did you have before?
   1. Why did you change to Port-a-Cath?
2. Have you followed the guidelines form The Cystic Fibrosis Center West at Aarhus University Hospital Skejby?
3. Have you had bolus injection, infusion pump or bolus?
4. Have you experienced any complications?
   1. Infection
   2. No return flow
   3. Occlusion
   4. Pain
   5. Other problems
5. Does the appearance of the Port-a-Cath bother you?
6. Have you experienced any problems with mixing the medicine if that has been necessary?
   1. If yes, do you think that it takes a long time to mix?
7. Have you had problems with the medicine storage?
   1. Do you think that it takes op a lot of space?
